# Balance Between Tooth Size and Tooth Number Is Controlled by Hyaluronan

**DOI:** 10.3389/fphys.2020.00996

**Published:** 2020-08-24

**Authors:** Natalia Sánchez, María Constanza González-Ramírez, Esteban G. Contreras, Angélica Ubilla, Jingjing Li, Anyeli Valencia, Andrés Wilson, Jeremy B. A. Green, Abigail S. Tucker, Marcia Gaete

**Affiliations:** ^1^Department of Anatomy, Faculty of Medicine, Pontificia Universidad Católica de Chile, Santiago, Chile; ^2^Faculty of Medicine, Universidad de Chile, Santiago, Chile; ^3^Centre for Craniofacial and Regenerative Biology, King's College London, London, United Kingdom

**Keywords:** successional tooth development, cell orientation, organogenesis, activator-inhibitor, molar development

## Abstract

While the function of proteins and genes has been widely studied during vertebrate development, relatively little work has addressed the role of carbohydrates. Hyaluronan (HA), also known as hyaluronic acid, is an abundant carbohydrate in embryonic tissues and is the main structural component of the extracellular matrix of epithelial and mesenchymal cells. HA is able to absorb large quantities of water and can signal by binding to cell-surface receptors. During organ development and regeneration, HA has been shown to regulate cell proliferation, cell shape, and migration. Here, we have investigated the function of HA during molar tooth development in mice, in which, similar to humans, new molars sequentially bud off from a pre-existing molar. Using an *ex vivo* approach, we found that inhibiting HA synthesis in culture leads to a significant increase in proliferation and subsequent size of the developing molar, while the formation of sequential molars was inhibited. By cell shape analysis, we observed that inhibition of HA synthesis caused an elongation and reorientation of the major cell axes, indicating that disruption to cellular orientation and shape may underlie the observed phenotype. Lineage tracing demonstrated the retention of cells in the developing first molar (M1) at the expense of the generation of a second molar (M2). Our results highlight a novel role for HA in controlling proliferation, cell orientation, and migration in the developing tooth, impacting cellular decisions regarding tooth size and number.

## Introduction

During development, the extracellular matrix plays a crucial role in controlling the growth and size of diverse organs (Jafari et al., 2010; Walker et al., 2018). Carbohydrates are important components of this matrix; however, their role is poorly studied in developmental biology. Hyaluronan (HA), also known as hyaluronic acid, is a polymeric carbohydrate and a major component of the extracellular matrix. HA is distributed widely throughout the connective and epithelial tissues and can absorb large quantities of water, regulating the swelling pressure for tissue rigidity and biomechanical integrity (Toole, 2001; Spicer and Tien, 2004). Apart from this structural role, HA can interact with cell-surface receptors and activate the HA signaling pathway. CD44 is the most common HA receptor and its activation regulates cell aggregation, proliferation, survival, and migration (Lee and Spicer, 2000; Bourguignon et al., 2007; Pure and Assoian, 2009; Senbanjo and Chellaiah, 2017; Chen et al., 2018). HA is particularly abundant in tissues that proliferate and grow during embryogenesis, regeneration, and carcinogenesis, as it provides the conditions for cell migration and proliferation, thereby promoting tissue remodeling (Chen and Abatangelo, 1999; Jiang et al., 2007; Contreras et al., 2009; Preston and Sherman, 2011).

HA is synthesized by hyaluronan synthases (HAS), which are located at the plasma membrane, and extrude HA into the extracellular space (Weigel, 2015; Baggenstoss et al., 2017). In mammals, there are three HAS. Among these, HAS1 and HAS2 synthesize high molecular weight (HMW) HA, while HAS3, the most active of the three enzymes, polymerizes low molecular weight (LMW) HA (Spicer and Nguyen, 1999; Itano and Kimata, 2002; Tammi et al., 2011; Vigetti et al., 2014). *HAS2* null mutant mice show cardiac defects and die at E9.5 (Camenisch et al., 2000), whereas *HAS1* and *HAS3* single or double mutations are viable (Bai et al., 2005; Kobayashi et al., 2010; Kessler et al., 2015). In these knockouts, HAS2 levels increase and appear to compensate for loss of *HAS1* and *HAS3* (Wang et al., 2014).

Tooth development is an excellent model to understand cellular dynamics (proliferation, cell movement, and cell shape, etc.) during embryogenesis. The autonomy of these processes enables explants to faithfully replicate normal development (Alfaqeeh and Tucker, 2013; Gaete et al., 2015). The formation of a tooth begins by an oral epithelium thickening called the dental placode, which then forms a bud (bud stage) while the subjacent mesenchyme condenses around it and the epithelium, transitions to form a cap shape (cap stage) and then a bell (bell stage), where histodifferentation starts. From the cap stage, the dental epithelium is divided into the outer dental epithelium, the stellate reticulum in the center, the stratum intermedium, and the inner dental epithelium. The inner dental epithelium forms the ameloblasts for enamel production, while the mesenchyme inside the enamel organ forms the dental papilla, from which the external layer forms odontoblast for dentin formation (Tucker and Sharpe, 2004; Nanci, 2017).

In humans and mice, a single molar dental placode gives rise to three successional molars in each jaw quadrant. At E13.5, the first molar (M1) has reached the bud stage with a molar “tail” starting to develop at the posterior edge of the M1 (Gaete et al., 2015). At E14.5, the second molar (M2) starts to develop within the molar tail. Finally, the third molar (M3) forms postnatally from the posterior edge of the M2 (Chlastakova et al., 2011). When the molar placode is dissected out and cultured *in vitro*, the molar dentition forms in its normal sequence, highlighting the fact that all three molars form from this primordium (Lumsden, 1979; Gaete et al., 2015).

HA and the components of the HA pathway are expressed in dental tissues during embryogenesis and decrease after birth, suggesting a role during tooth development (Felszeghy et al., 2000, 2001, 2005; Tien and Spicer, 2005; Yang et al., 2016). HA is present in the dental epithelium and basal membrane during the bell stage, and it is localized around secretory cellular projections in ameloblasts and odontoblasts (Felszeghy et al., 2000). HA is also observed in the intercellular spaces in the stellate reticulum of the dental organ as these intercellular spaces expand (Felszeghy et al., 2000). According to chromatographic analyses, HA is the main and largely the only glycosaminoglycan present in dissociated dental epithelium, whereas in the mesenchyme HA, heparan and chondroitin sulfate proteoglycans are present (Lau and Ruch, 1983). HAS1 and HAS2 are expressed in the dental epithelium and mesenchyme, but their expression decreases over time, while HAS3 is mainly detected in the core of the dental epithelium (Tien and Spicer, 2005; Morita et al., 2016; Yang et al., 2016) and its expression increases over time (Yang et al., 2016). CD44 is found in the oral epithelium, dental lamina, and stratum intermedium, and moderately in the stellate reticulum (Nakamura and Ozawa, 1997; Felszeghy et al., 2001).

Although the presence of HA and its related components have been described during tooth development, its role is unknown. It has been demonstrated that the general inhibition of the synthesis of gycosaminoglycans inhibits tooth development (Jiang et al., 2019), however the specific role of HA was not studied. Here, we evaluated the functional role of HA during successional molar development in the mouse, characterizing the expression of the components of the HA pathway at early stages of dental development and performing loss-of-function approaches by blocking HA synthesis in tooth germ culture using a chemical inhibitor. We show that, when the production of HA is inhibited, the budding of a new molar is blocked, and the first molar increases in size. These changes in size and number appear to be controlled by alterations in cell orientation and proliferation. HA, therefore, plays an unexpected and novel role in regulating the balance between tooth size and tooth number. These findings provide important insights into how organ size can be regulated.

## Materials and Methods

### Animals and Explant Culture

All experimental procedures were performed following the requirements and approval of Pontificia Universidad Católica de Chile and the King’s College London Ethics Committees. Mouse embryos were collected at E14.5 to perform cultures. Experiments were performed in wild type CD1 and/or C57BL/6J according to the availability of the strains. Figures show CD1 data. Supplementary Figure S1 show C57BL/6J data. For cultures, we used the air-liquid interface method: dissected mandibular molar placodes were placed on top of transparent nucleopore filters (VWR) supported by metal grids at the surface of Advanced DMEM/F12 culture medium (Gibco®), supplemented with 1% penicillin/streptomycin (Gibco®) and 1% GlutaMAX®. These cultures were incubated at 37°C/5% CO_2_, and the medium was changed three times per week (Alfaqeeh et al., 2013; Gaete and Tucker, 2013; Gaete et al., 2015). The cultures were treated with 200 μM 4-methylumbelliferone (4-Mu; a chemical inhibitor for HA synthesis, Sigma-Aldrich M1381) from a of 200 mM stock solution in DMSO, while DMSO was added to control cultures. This concentration of 4-Mu has previously been shown to cause inhibition of tail regeneration in *Xenopus laevis* tadpoles (Contreras et al., 2009) and was chosen after a titration in our system. Experimental and control pairs were obtained using contralateral molar regions.

### Whole Mount Immunofluorescence, F-Actin Staining and Fate Mapping

Whole-mount immunostaining was performed as described (Gaete and Tucker, 2013), with minor modifications: permeabilization and washes were performed using PBS-Tr 1% (1% Triton X-100 in 1X phosphate-buffered saline). The following antibodies and dilutions were used: anti-phospho-Histone H3 at 1:300 (Merck, 05-806) and CD44 1:25 (5D2-27 supernatant, Developmental Studies Hybridoma Bank). Alexa Fluor® 488 or 568 (Thermo Fisher) at 1:500 was used as secondary antibodies. For F-actin and DNA staining, fixed samples were washed in 1X PBS and incubated overnight at 4°C with Phalloidin Alexa Fluor 488 (Thermo Fisher) at 1:150 in 1% BSA in PBSTr 0.5% (0.5% Triton X-100 in 1X PBS). In all samples, DNA was stained with Hoechst 33342 dye (Sigma-Adrich), washed in PBS for 2 h, mounted with Fluoroshield™ (Sigma-Aldrich), and scanned using a Leica SP5 confocal microscope. For fate mapping, DiI [CellTracker CM-DiI (C7000, ThermoFisher)] was re-suspended in 100% ethanol, and dried crystals at the tip of a tungsten needle were placed into the molar region using a micromanipulator. The position of the DiI label was imaged during the culture period to follow cell movement.

### Histology and *in situ* Hybridization

Samples were fixed in 4% PFA at 4°C, washed in 1X PBS/DEPC, incubated in methanol series, isopropanol, cleared in tetrahydronaphtalene, embedded in paraffin, and sectioned at 8 μm in a microtome. For histology, samples were rehydrated and stained with Mayer’s hematoxylin, 1% alcian blue 8GX (Merck, 33864-99-2), and alcoholic eosin Y, dehydrated and mounted in mounting media (Cancer Diagnostics). For *in situ* hybridization, Has2 and Has3 plasmids that contain EGFP in their N-terminus were kindly provided by Dr. Markku Tammi, and originally came from Dr. Andrew Spicer, being completely functional as to hyaluronan synthesis (Rilla et al., 2005; Kultti et al., 2006). We amplified and subcloned the complementary DNA (cDNA) in pBluescript SK plasmid to generate HAS2 and HAS3 probes. Digoxigenin-labeled RNA probes were synthesized using T7, T3, and Sp6 RNA polymerases (Roche). 10 μm sections were rehydrated, permeabilized in 10 μg/ml proteinase K for 15 min, refixed in 4% PFA for 20 min, and incubated in hybridization solution: 1 μg/ml *HAS2*, or *HAS3* probes in hybridization buffer (50% formamide, 20 mM Tris/DEPC pH 7.5, 300 mM NaCl/DEPC, 5 mM EDTA/DEPC, 1x Denhardt’s solution, 10% dextran sulfate, and 0.5 mg/ml tRNA) overnight at 65°C. Samples were washed in 50% formamide/2x SSC, 2x SSC, and 0.2x SSC, each wash performed twice for 30 min at 60°C. Then, sections were washed in Maleic Acid Buffer plus Tween (MABT) and blocked in blocking buffer: 10% of goat serum Gibco® and 1% Boehringer Blocking reagent (BBR), in MABT buffer for 2 h at room temperature and incubated with 1:1000 anti-DIG Alkaline Phosphatase antibody (Roche) in blocking buffer overnight at 4°C. Samples were washed four times in MABT buffer for 30 min and incubated twice in AP-Buffer (100 mM Tris-HCl pH 9.5, 50 mM MgCl2, 100 mM NaCl, and 0.1% Tween 20) for 10 min. The color reaction was developed using BM purple (Roche).

### Hyaluronan Detection

For HA detection, paraffin sections, obtained as described above, were cleared and rehydrated to 1x PBS. Endogenous peroxidase was blocked using 0.3% H_2_O_2_ in cold methanol for 15 min on ice. Samples were incubated with 1:200 hyaluronic acid binding protein (HABP, Biotinylated, Merck) in 1% BSA at 4°C overnight. Then, samples were washed and incubated with ABC kit (Vectastain Elite Standard, Vector Labs) for 30 min at room temperature and revealed with DAB reaction (DAB substrate peroxidase kit, SK 4100, Vector Labs), dehydrated and mounted with mounting media (Cancer diagnostics). For fluorescent HA detection, whole fixed slices of tissue were fixed in 4% PFA, permeabilized 45 min in PBSTr 1% (1x PBS, 1%Triton X-100), incubated with 1x trypsin (Gibco®) on ice for 15 min, washed and blocked with 10% goat serum, 1% DMSO, and 1% Triton X-100 for 2 h at room temperature, and incubated with 1:100 HABP in the blocking solution overnight at 4°C. The samples were washed with PBSTr 1% and incubated with ABC kit for 1 h at room temperature, then the samples were incubated with 1:500 anti-streptavidin Alexa Fluor™ 488 (Thermo Fisher) and 1:1000 Hoechst overnight at 4°C. Finally, the samples were washed in PBSTr 1% and mounted with Fluoroshield™ (Sigma-Aldrich).

### Image Capture, Processing, and Statistics

Slices and placodes cultures were photographed during the culture period using a Nikon SMZ18 Stereomicroscope. Immunofluorescence, fluorescence detection of HA, phospho-Histone H3 (pH3), and DiI labeling was scanned on a confocal microscope (Leica SP5 laser scanning confocal microscope). Images were processed using Fiji software and Adobe Photoshop CC. For CD44, autofluorescence subtraction was applied on Fiji. For live explant culture photography, Smart Sharpen filter was applied to eliminate Gaussian liquid effect. To obtain the area and growth rate of the cultures, the contour tooth germs or from the stereomicroscope images were delimited and the area calculated at different days using Fiji. A ratio between the enamel knot and the entire dental region was calculated. The shape and the absence of proliferation were used as a guide to observe the enamel knot and the total germ using pH3 immunostained samples. The areas were plotted and analyzed by Student’s *t*-test using GraphPad Prism 5 software. For growth measurements, sequential histological sections stained as described before, were photographed and the molar area was delimited, drawn, and filled using Adobe Photoshop CC and calculated using Fiji software. Volume was calculated as the area under the curve using GraphPad Prism 5 software. To obtain the mitotic index, the percentage of pH3 positive cells under total cells was calculated. To differentiate the anterior and posterior proliferation, the M1 was virtually divided in anterior and posterior half, pH3+ cells were calculated and area was measured finally, and pH3+ cells per area was determined. For cell density calculations, the total cells of the tooth germ were manually counted in four Z-level plane sections in four independent explants pairs (*n* = 8), in the entire germ, and divided by the area occupied in mm^2^. No significant differences between the different zones of the tooth were found. Results were plotted and analyzed by Student’s *t*-test using GraphPad Prism 5 software.

### Cellular Shape and Orientation Analysis

Confetti transgenic mice that express fluorescent proteins after Cre excision were utilized to follow cell clones (Schepers et al., 2012). Pregnant female mice carrying R26R-CreER/Confetti embryos were injected with 1.5 mg tamoxifen at E13.5. This amount of tamoxifen was the minimum needed to obtain labeling of single cells or small clones at the end of the culture period. The treated embryos were collected at E14.5 and the molar region was cultured, treated with 4-Mu (200 μM) or DMSO as control for 2 days, then scanned using multiphoton microscopy (Zeiss 7MP). The images provided were analyzed by 3D segmentation and ellipsoid fitting method in the cell membrane expressed CFP using Fiji software and associated plug-ins, providing information to investigate cell clones, shape, volume, orientation, and major axis of four samples from three litters (total *n* = 33 cells per group). To determine the cell shape, ratios between R1/R2, R1/R3, or R2/R3 were obtained. The ratio of the radius of principal and secondary axes of ellipsoids ranging from 0.5 to 2, was calculated and values near to 1 indicate a rounded cell. The data were plotted and analyzed by Student’s *t*-test using GraphPad Prism 5 software. To determine the axis orientation of the cell, the angle formed between the ellipsoid and the XY, XZ, and YZ planes of the molar was determined. The angles were plotted in a rose-type graph using Oriana4 software in categories of 15° each and statistical analysis to measure distribution of ordinal data was made using Mann-Whitney-Wilcoxon test.

## Results

### Components of the Hyaluronan Pathway Are Expressed During Molar Placode Development

**Figure 1:**
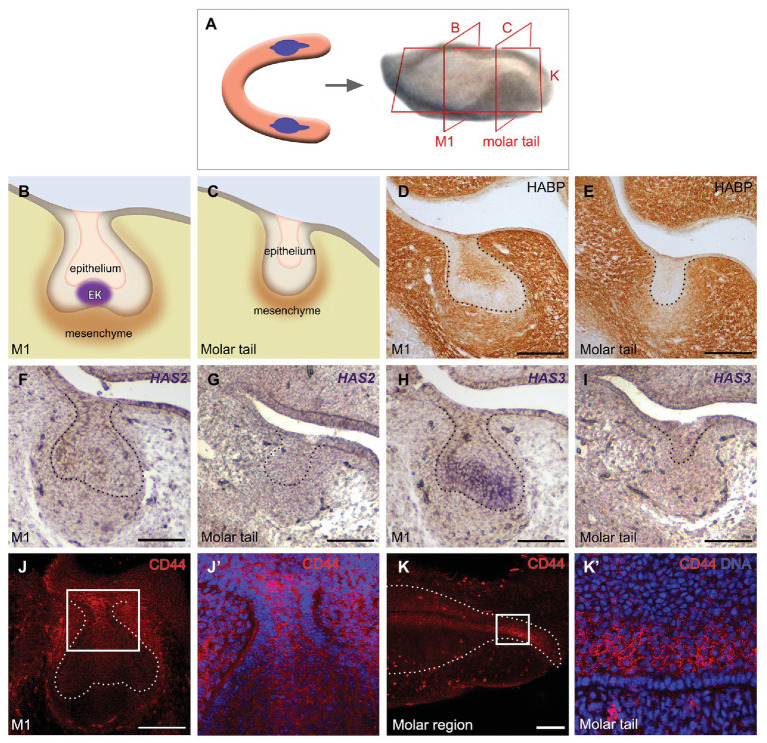
Components of the hyaluronan (HA) pathway are detected in the molar germ. **(A–C)** Schematics of the **(A)** developing molar region and **(B)** a section at the level of **(B)** M1 and **(C)** the molar tail. **(D,E)** Detection of HA by hyaluronic acid binding protein (HABP) in the dental mesenchyme and epithelial core of the **(D)** molar and less intense in the **(E)** tail. **(F–I)** *In situ* hybridization of HA synthase 2 and 3 (*HAS2* and *HAS3*), **(F)** HAS2 is expressed in the lingual oral epithelium and weakly in the dental mesenchyme, whereas **(H)** HAS3 is detected mainly in the stellate reticulum, inner epithelium, and enamel knot. **(G,I)** No expression of *HAS2* or *HAS3* was evident in the molar tail. **(J,K)** CD44 immunofluorescence in a **(J)** frontal view of the molar region, including a **(J’)** zoom of the region indicated in **(J)** and **(K)** in a sagittal view at M1 including a **(K’)** zoom of the region indicated in **(K)**. CD44 is expressed in complementary regions together with *HAS3*. Scale bar: 100 μm.

To understand the role of HA during molar development, we first analyzed the presence of HA in the molar region at E14.5 at the level of the first molar germ (M1; Figures 1A,B) and in the molar tail, a region posterior to the M1 where the successional molar 2 germ (M2) will develop (Figures 1A,C). For specific HA localization in the molar germ we used biotinylated HABP, which demonstrated that HA is present throughout the dental mesenchyme (Figures 1D,E). Within the epithelium, HA was present mainly in the stellate reticulum at the center of the M1 (Figure 1D) and weakly in the middle epithelium of the molar tail (Figure 1E). The stellate reticulum and middle epithelium are characterized by star-shaped cells surrounded by extensive intercellular spaces. The condensed dental mesenchyme stained less intensely than the rest of the mesenchyme (Figures 1D,E). Thus, HA was localized in the developing molar mesenchyme and in the core of the epithelium.

This result prompted us to identify where HA is synthesized in the molar placode in our system, following on from previous reports (Tien and Spicer, 2005; Morita et al., 2016). By *in situ* hybridization, we observed that the mRNA of *HAS2* and *HAS3* was expressed in the E14.5 M1. *HAS2* was detected in the lingual oral epithelium and weakly in the mesenchyme (Figure 1F). Within the epithelium, *HAS3* was expressed in the stellate reticulum, inner enamel epithelium, and enamel knot (Figure 1H), agreeing with previously reported expression patterns (Tien and Spicer, 2005). No expression of *HAS2* or *HAS3* was evident in the molar tail (Figures 1G,I). The HA receptor CD44 was detected in the more superficial epithelium connecting the M1 to the oral epithelium and in the middle dental epithelium of the molar tail by immunofluorescence (Figures 1J–K’). Thus, HA is widely expressed in the tooth, while HAS3 and CD44 have almost complementary expression in the inner enamel epithelium, while only CD44 is present in the epithelium of the molar tail.

### Hyaluronan Synthesis Inhibition Increases the Size of the Developing Molar Germ, but Decreases the Formation of the Successional Molar

**Figure 2:**
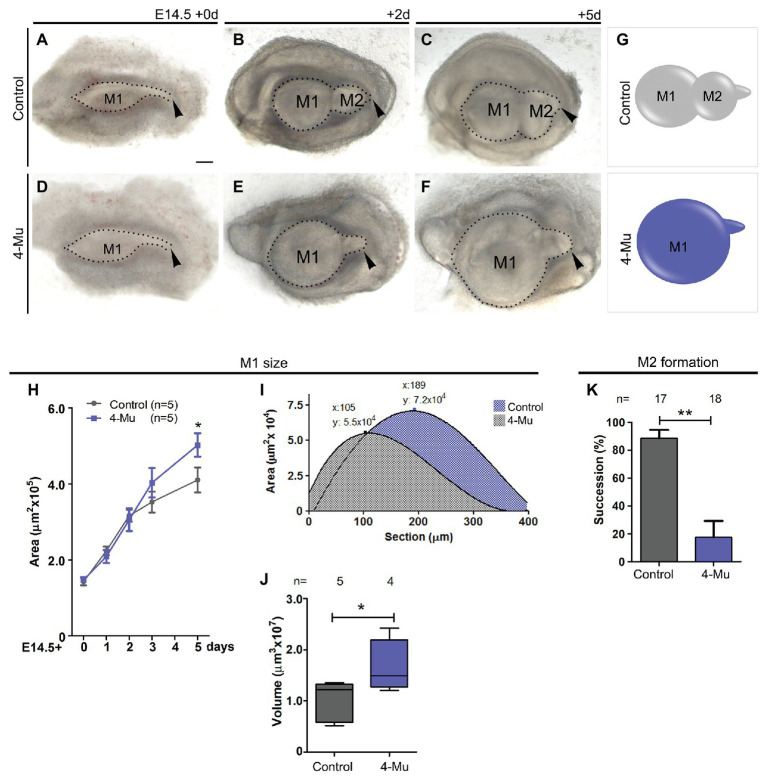
Inhibition of hyaluronan synthase (HAS) generates a big tooth germ and impairs the formation of the second molar. **(A–F)** Molar placodes were dissected from an E14.5 embryo mandible and cultured in control **(A–C)** or 4-methylumbelliferone (4-Mu; **D–F**) media until 5 days. 4-Mu treated cultures show a bigger M1 and no M2 formation comparing to control culture. Arrowhead indicates the tail of the molar placode where the next tooth should be formed. **(G–J)** First molar growth in control and 4-Mu treated cultures. **(G)** Scheme indicating the M1, whose **(H)** area was measure showing that treated cultures, generate significant bigger teeth. **(I)** Area under the curve of M1 was calculated confirming the increase of volume of the 4-Mu treated placodes. **(J)** Box-plot of the M1 volume shows almost two times of increase in the volume of 4-Mu treated placodes. **(K)** M2 successional molar formation in percentage, showing a decrease of almost four times of the presence of M2 in 4-Mu treated culture. Scale bar: 100 μm. ^*^p < 0.05; ^**^p < 0.01.

To assess the function of HA during molar formation, we performed explant cultures in the presence of 4-Mu, a chemical compound that specifically inhibits HA synthesis (Nakamura et al., 1995; Kakizaki et al., 2004). Molar placodes from E14.5 mouse embryo were dissected out and cultured in control medium (Figures 2A–C) or in the presence of 4-Mu (Figures 2D–F). A reduction of HA was observed in treated cultures (Supplementary Figure S1). After 2 days, the first and the second molar germ had formed in control cultures (Figure 2B), whereas in the 4-Mu treated cultures, the M1 appeared bigger than the control culture, while the M2 did not develop (Figure 2E). After 5 days of culture, a large M1 was observed in the 4-Mu treated group (Figure 2F) in comparison to control cultures (Figure 2C). The inhibition of the formation of the M2 was maintained after 5 days of culture (Figure 2F, arrowhead). We quantified the increase of the M1 area at different days post-culture. At 3d, the 4-Mu treated cultures started to grow more than controls, and at 5d appeared significantly larger than controls (Figures 2G,H). At the end of the culture period, the molars were processed for volume analysis, confirming that 4-Mu treated M1 were significantly larger than controls (Figures 2I,J). Changes in the size of the molar could be related to a change in the size of the enamel knot. A ratio between the size of the enamel knot and the tooth germ was calculated in the cultured samples (Supplementary Figure S2A). We found non-significant differences in the relative size of the enamel knot between control and treated conditions after 2 days of culture, meaning that, as the molar germ grew in size, the enamel knot is also expanded in proportion. Moreover, *in situ* hybridization against *Shh* on explants cultured for 5 days, showed no difference in the pattern of expression (data not shown).

To analyze the molar succession, we quantified the number of samples that developed M2 at day 3 of culture in the presence of 4-Mu. We found that the formation of M2 occurred in less than 20% of the 4-Mu-treated cultures (*n* = 4/18), whereas the M2 was well-developed in the majority of control cultures after 3 days (*n* = 14/17; Figures 2G,K). Altogether, the use of a HA synthesis inhibitor produced an opposing phenotype among the molars: the developing M1 became bigger, while the formation of the M2 was inhibited.

### 4-Mu Treatment Increases Cell Proliferation of the Developing Molar

**Figure 3:**
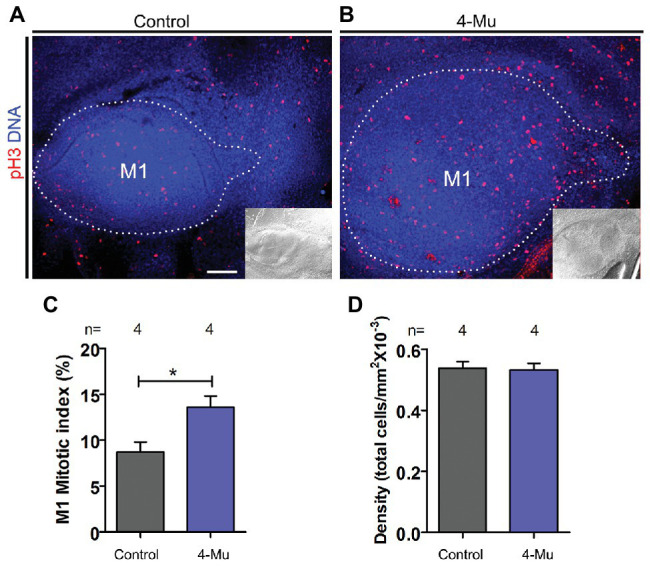
Proliferation is increased in 4-Mu treated placodes. **(A,B)** Immunofluorescence of pH3 of **(A)** control and **(B)** 4-Mu treated placodes showing an increase of the proliferation in 4-Mu cultures. An inset of the DIC channel is included for a general view. **(C)** Mitotic index of M1 in control and 4-Mu treated cultures indicating a 5% of increase in 4-Mu cultures. **(D)** M1 cell density shows no difference between control and 4-Mu treated cultures. Scale bar: 100 μm. ^*^p < 0.05.

In order to find a cellular mechanism for the increase in size of M1 after HA synthesis inhibition, we investigated whether cell proliferation was altered after 4-Mu treatment. By immunofluorescence detection of the mitotic marker pH3, we observed an increase in the proportion of proliferating cells in the dental organ and a significant increase in the mitotic index in 4-Mu treated cultures compared to controls (Figures 3A–C), while the cell density was conserved (Figure 3D). The distribution of the proliferation was similar in the anterior and posterior compartments when compared (Supplementary Figure S2B). These results indicate that the HA-inhibited M1 is bigger than controls because of an increased cell proliferation, but without affecting cell density (*n* = 4).

### Hyaluronan Is Necessary to Conserve the Morphology of the Molar Tail

**Figure 4:**
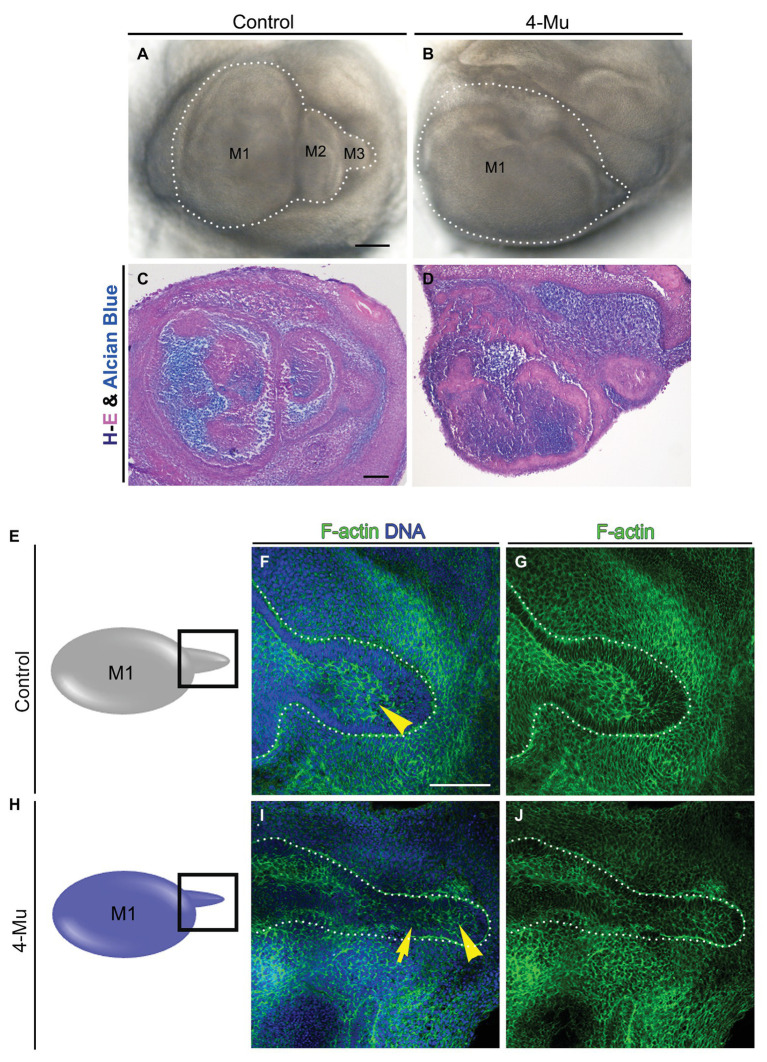
Morphology of tooth germ is conserved in 4-Mu treated cultures, but not in the tail **(A,B)** molar placodes cultured by 5 days in **(A)** control or **(B)** 4-Mu conditions, showing the increase of size and the lack of succession in 4-Mu treated cultures. **(C,D)** Trichrome staining of molar placode sections showing no differences in morphology in **(C)** control or **(D)** 4-Mu cultures. **(E–J)** Morphology of molar tail in **(E–G)** control and **(H–J)** 4-Mu treated cultures visualized by F-actin and DNA staining showing a thinner and disorganized molar tail in 4-Mu cultures. The middle epithelium is not distinguishable in 4-Mu cultures (yellow arrowhead) and less stratification in the epithelium is observed (yellow arrow). Scale bar: 100 μm.

To determine whether the larger tooth germs generated by 4-Mu treatment had differences in histomorphology or organization of the tissues, we used conventional hemotoxylin-eosin and alcian blue staining. No key differences between the tissue of the control and the 4-Mu treated molar germs were observed (Figures 4A–D). As molar succession was impaired, we analyzed the morphology of the molar tail and its proliferation. Using F-actin and DNA staining, we observed that in the 4-Mu treated explants, the molar tail was thinner and with fewer layers in contrast to untreated explants, in which the tail was bigger, stratified and with a distinguishable middle epithelium (Figures 4E–G compare to Figures 4H–J, seen in 4/4 experimental samples, compared to 2/2 controls). However, tail proliferation was increased (Supplementary Figure S2C). In conclusion, loss of HA synthesis conserved the general morphology of the M1; however, it disrupted the size and structure of the tail.

### Altered Dental Cell Shape and Orientation Is Observed After Blocking Hyluronan Synthesis

**Figure 5:**
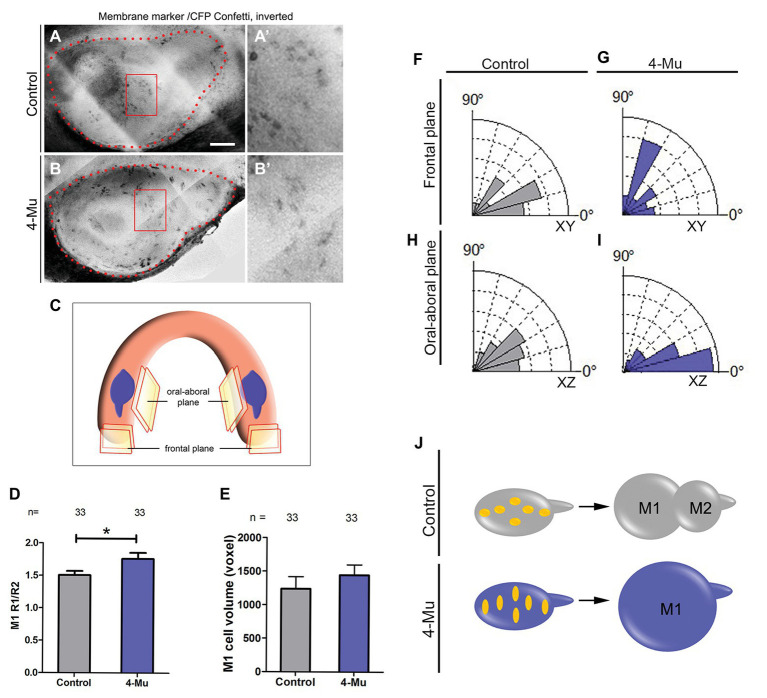
4-Mu treated cultures show more elongated and oral-aboral orientated cells compared to control. **(A,B)** Z-projection of CFP-label **(A,A’)** control and **(B,B’)** 4-Mu molar placodes analyzed. **(A’,B’)** are magnifications from **(A,B)**, respectively. **(C)** Mandible scheme showing the molar placodes and the orientation planes analyzed by multiphoton microscopy from Confetti mouse molars. CFP-label (cell membrane marker) was analyzed to determine **(D)** cell shape, **(E)** M1 cell volume, and **(F–I)** orientation in control and 4-Mu treated cultures. **(D)** Ratio between two radiuses of the cells showing that 4-Mu cultures have more elongated cells. **(E)** M1 cell volume showing no differences between control and 4-Mu treated samples. **(F–I)** Cell orientation in frontal plane of **(F)** control and **(G)** 4-Mu treated placodes and oral-aboral plane of **(H)** control and **(I)** 4-Mu cultures, indicating that 4-Mu cells were aligned toward the oral-aboral axis (see **A** for planes), while control cells were orientated in the antero-posterior axis. The significance of the distribution of the uncategorized angles was ^*^XY and ^**^XZ. **(J)** Scheme of cell shape and orientation in control and 4-Mu treated cultures. Scale bar: 100 μm. ^*^p < 0.05; ^**^p < 0.01.

After blocking HA synthesis, an increase in M1 cell proliferation and size occurred together with a formation of a smaller tail. This phenotype could be explained by a defect in cell orientation and movement. Therefore, we evaluated cell shape and orientation after treatment with 4-Mu. For this, we used Rosa26-Cre:Confetti transgenic embryos (Schepers et al., 2012) to trace cells in the molar placode. This transgenic mouse expresses fluorescent proteins after Cre excision. Pregnant female mice were injected with the minimum of tamoxifen to obtain single cell resolution *in vivo* and after 24 h, and the developing molars of their embryos were cultured in the presence of 4-Mu for 2 days (Figures 5A,B). We analyzed the plasma membrane fluorescent marker (CFP) in two planes to understand cellular shape and orientation (Figure 5C). We found that after 2 days of 4-Mu treatment, cells were significantly more elongated than cells in control cultures (Figure 5D). Additionally, the cells from 4-Mu explants displayed an orientation closely aligned toward the oral-aboral axis, while in control cultures cells were more rounded and oriented in the antero-posterior axis (Figures 5F–I, see schemes of the planes and cells in Figures 5A–J) with significant differences. Therefore, control M1 cells were mildly elongated in an axis toward the M2, while in the 4-Mu treated explants, cells were strongly elongated in an axis toward the top of the M1, losing their normal orientation (Figure 5, see summary in Figure 5J). Altogether, our results indicate defects in cell shape and orientation after impairing HA synthesis.

### Hyaluronan Is Required for Cell Migration During Molar Development

**Figure 6:**
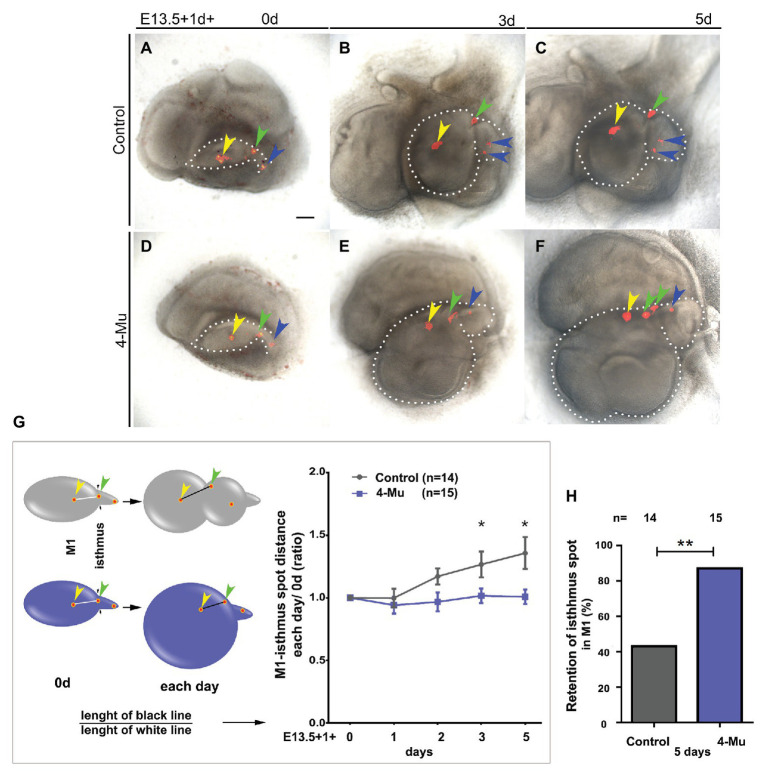
Cell movement is affected in 4-Mu treated cultures. **(A–F)** DiI labeling was used to label three areas along the **(A–C)** control and **(D–F)** 4-Mu treated molar placode at E13.5 from anterior to posterior regions (yellow, green, and blue arrowhead, respectively). Placodes were cultured for up to 5 days and the position of the label followed. **(A,D)** day 0. **(B,E)** day 3. **(C,F)** day 5. **(G)** In 4-Mu cultures the label in the M1 and in the isthmus (middle label, indicated by the green arrowhead) retained a fairly constant relative position, while in controls comparable labels moved apart after 3–5 days in culture. **(H)** The isthmus label was retained in M1 in most of the 4-Mu treated cultures. Data based on *N* = 14 control and 15 treated placodes. Scale bar: 100 μm. ^*^p < 0.05; ^**^p < 0.01.

Given that the M2 is formed at the tail region of the molar placode, we asked whether this phenotype is due to retention of some cells in the region of the first molar. To answer this question, we performed lineage-tracing experiments. We used DiI to label the M1 and the tail of the molar placode in three dots from anterior to posterior regions (Figures 6A,D, indicated by the yellow, green, and blue arrowhead, respectively) after 1d starting with E13.5 molar placodes. This timing allowed the tissue to stick to the filter for a precise labeling. We followed the labels and after 3–5 days in culture, observing that the dots were closer in the 4-Mu treated cultures compared to the control cultures, in which the dots were more separated and scattered within the molar tissue (Figures 6A–F). To quantify this, we divided the distance between the anterior dot and the middle dot after culture by the original distance at day 0. The middle dot (green arrow) was named the “isthmus spot,” because it was located at the posterior edge of M1 and the beginning of the molar tail. As suggested by the images, the dots between the center of the M1, and the isthmus had moved significantly further apart when compared to the 4-Mu treated cultures (Figure 6G). In the displayed culture (Figures 6A–F), we observed that part of the isthmus spot that contributes to the second molar in the control, was recruited into the first molar (Figures 6C,F, green arrow). The isthmus spot fate was therefore followed throughout the culture period and the final position registered. In the treated cultures, approximately 90% of the samples retain the isthmus dot in M1 compared to approximately 40% in the controls (Figure 6H). This indicated a lack of movement of cells from the first molar into the second molar after the treatment with 4-Mu, resulting in cells that normally contributed to the second molar, were recruited into the first molar.

## Discussion

In this work, we studied the function of HA during successional molar development in mice. For this, the molar region was exposed to an inhibitor of HA synthesis when the M1 was at the cap stage and the M2 was forming from the tail of the M1. Interestingly, the phenotype that we observed between the two molar germs was paradoxical: the M1 continued its development and increased its size as M2 was arrested in its formation.

Our results indicate that the inhibition of HA synthesis caused an increase in the mitotic index of the M1 cells, explaining the size of the M1. The increase in proliferation after the incubation with 4-Mu in our system is at odds to the findings published in other systems, in which decreasing HA synthesis reduced cell proliferation (Contreras et al., 2009; Saito et al., 2013; Wang et al., 2015; Nagase et al., 2017; Ouyang et al., 2017). Similarly, this phenotype is in contrast to the reduced proliferation and molar size reported after inhibition of the synthesis of general glycosaminoglycans (Jiang et al., 2019). This suggests that the effect might be stage dependent, or that there is compensation from other glycosaminoglycans after HA inhibition.

**Figure 7:**
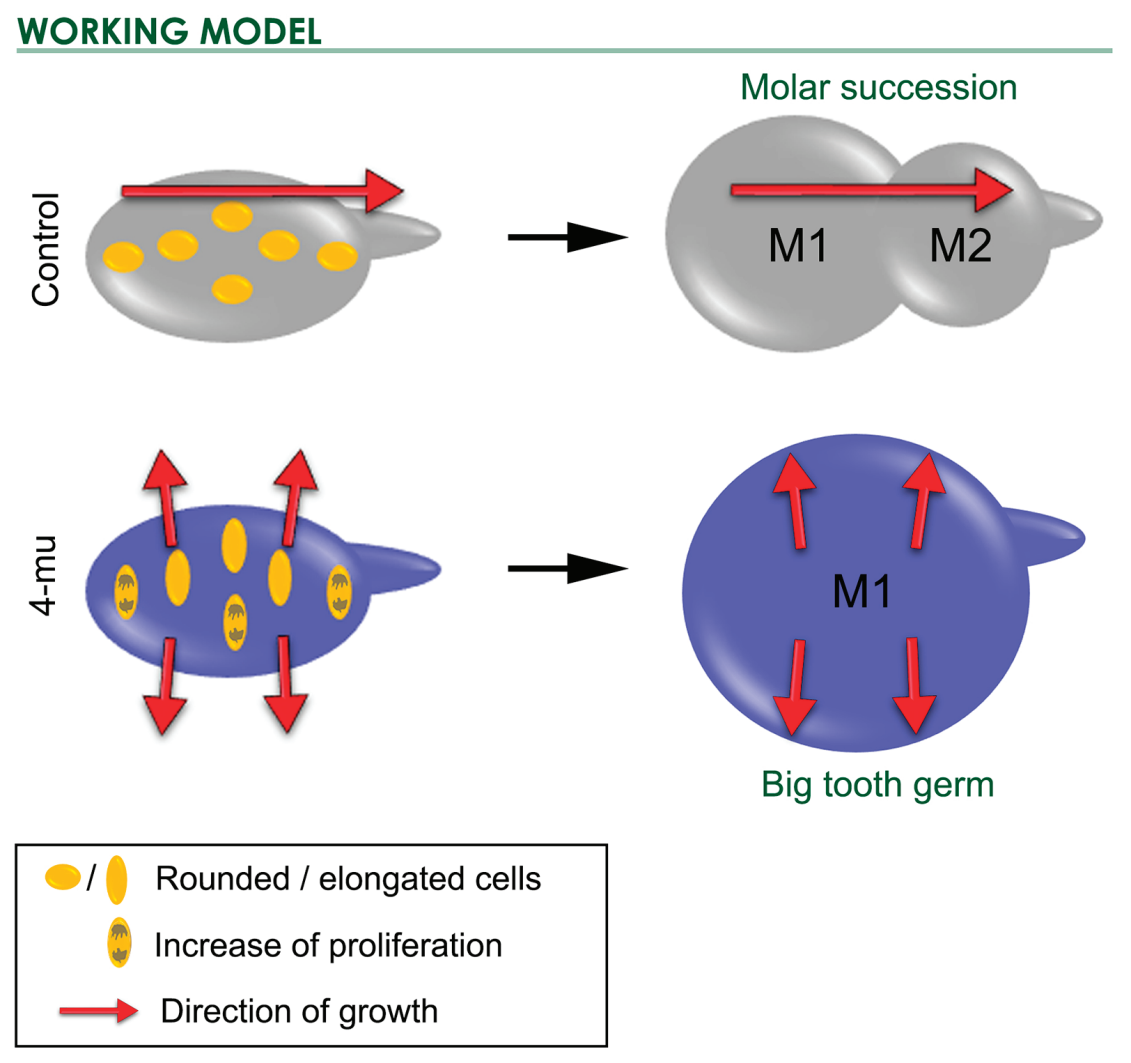
Working model. Scheme of control and 4-Mu treated cultures *at initio* and after 5 days of treatment. M1 cells were mildly elongated in an axis toward the M2, however in the 4-Mu, cells were strongly elongated in an axis that follows the expansion of M1, which proliferates more than the control, resulting in a bigger M1 and impairment in the formation of the successional M2.

In the case of molar succession, the 4-Mu treated M1 was bigger, but the M1 tail was smaller than in control cultures. Interestingly, just before the phenotype appeared, cells of the first molar were more elongated but not in a direction toward the molar tail as in their control counterparts. This was correlated with the fact that cells in the posterior edge of the M1 (isthmus spot) did not migrate to be incorporated into the second molar. If the M2 is formed by contribution of cells from M1 (Juuri et al., 2013), therefore, after inhibition of HA synthesis, some cells that normally would have migrated to form the M2 have now been retained in the field of the M1. Thus, the M1 gets bigger at the expense of the M2 (see working model in Figure 7). Although this model can certainly explain the observed phenotype, our results do not discard the possibility of an arrest in the development of the M2, as a consequence of a direct effect of HA on M2 development, independent of the effect on M1 growth. From our experiments, it is important to understand that we cannot distinguish whether the development of M2 is just delayed by 4-Mu or if it remains completely blocked. Thus, HA levels could interfere with the activator-inhibitor model, with the consequence that when M1 grows larger, its field of inhibition also increases and M2 fails to form. Later, M2 cells could be far enough from this field of inhibition, reactivating M2 development. Limitations of the culture system prevent yet longer culture. *In vivo* analysis using kidney capsule-cultures or mouse models such as *HAS3* knockout could provide the necessary time to address those questions.

At the start of the culture period, HA was shown to be present in the tooth mesenchyme and in epithelial tissues with mesenchymal or myxoid morphology, such as the stellate reticulum and the middle epithelium of the tail. Both the stellate reticulum and the middle epithelium have been proposed to be a source of stem cells in the mouse incisor (Wang et al., 2007) and in polyphyodont models (Smith et al., 2009) and express the transcription factor Sox9 (Gaete et al., 2015). HA may play a key role in the function of this tissue and the formation of a new tooth during molar succession, in addition to tooth replacement. Additionally, HA is involved in epithelial-mesenchymal transformation (Camenisch et al., 2000), which can be an event necessary for the epithelial migration to form M2 in the molar succession.

HA could also have a role on the formation of primary enamel knot. Our 4-Mu incubations started relatively late to understand initial patterning of the enamel knot (E14.5); therefore, early experiments should be conducted to address this possibility. Importantly, our results indicated that as the molar grow in size the enamel knot also increase in size, which means that overall tooth patterning could be proportionally enlarged as the phenotype appears.

Regarding its signaling properties, it has been shown that HA plays an important role during embryonic development regulating cell migration, shape, and proliferation (Spicer and Tien, 2004). *HAS3* is the most active enzyme of the HAS family and polymerizes shorter HA chains (Itano et al., 1999; Itano and Kimata, 2002) that stimulates cell signaling, proliferation, and epithelial-to-mesenchymal transition in cancer models (Kultti et al., 2014; Kuo et al., 2017; Arasu et al., 2019). *HAS3* expression is higher in the dental epithelium than the mesenchyme, with the distribution of CD44 appearing complementary to *HAS3* in the dental epithelium. This suggests that CD44 may mediate HA signaling in adjacent locations. It has been proposed that HA is synthesized in cells in the epithelial dental lamina to produce a pericellular HA coat, which may be required for the proliferation and migration of cells in the dental lamina into the underlying mesenchyme (Felszeghy et al., 2000). In our model, the assembly and presence of a pericellullar HA coat might facilitate the budding off for the successional molar formation.

The inhibition of HA synthesis did not impact the gross morphogenesis of the first molar, with normal formation of dental tissues. However, HA inhibition altered levels of proliferation and cell orientation. Interestingly, stimulated HA synthesis by HAS3 changes the cell polarization with aberrant mitotic spindle orientation in dividing cells that impact in epithelial thickness (Rilla et al., 2012).

Hydrogels can affect the diffusion of growth factors *in vitro* (Silva et al., 2009), and HA-CD44 interaction can affect the activation of growth factors in cancer (Misra et al., 2011). HA could have effects on the diffusion of both FGFs and Hhs during tooth development. Further investigation of these pathways, for example FGF targets, like Sprouty, and Hh targets such as Ptc and Gli, could be analyzed after 4-Mu treatment to address this question. This would allow us to clarify whether the phenotype observed was due to a change in signaling gradients or due to mechanical effects, which might drive cell rearrangement.

In this work, we considered that the effects of 4-Mu are due to the inhibition of HA-synthesis. 4-Mu is a highly specific inhibitor, and it is unlikely to affect any key pathways other than HA synthesis (Nakamura et al., 1995; Kakizaki et al., 2004; Kultti et al., 2009; Nagy et al., 2019). However, we cannot excluded the possibility that 4-Mu might be affecting other targets that control the sequential formation of molars. Having this in mind, any effects on alternative pathways could also be a consequence of the decrease in HA synthesis. Future directions analyzing the phenotype of *HAS* knockout animals would provide additional validation of the role of HA during molar formation.

It is known that the size and number of molar teeth that normally develop from the molar placode appears to be regulated by a system of positive signals from the mesenchyme and negative signals from the intermolar region (Kavanagh et al., 2007). The inhibitor model proposes M1 inhibits M2 development, and M2 inhibits M3. Thus, when the M2 primordium is removed from M1 in culture, the inhibitory signal is eliminated and M2 develops earlier than normal and reaches a greater size, depending on BMP signaling (Kavanagh et al., 2007). Hence, when a tooth reaches a given size the inhibition of the next tooth in the series is lost, allowing it to develop. This occurs efficiently when the original primordium is large (Cai et al., 2007). In our model, it is important to understand whether the development of M2 is just delayed by 4-Mu or whether it remains completely blocked in late phases. HA could interfere with the activator-inhibitor model, with the consequence that when M1 grows larger, its field of inhibition also increases and the M2 fails to form, unless the cells are far enough away from this field to be reactivated. *In vivo* analysis using kidney capsule-cultures or mouse models, such as *HAS2/HAS3* knockout animals, would provide the necessary temporality to address such questions. Moreover, the involvement of HA synthesis could be supported by directly blocking the expression of *HAS2* and/or *HAS3*.

In summary, our results suggest that HA has a pivotal role during tooth development, regulating the size of the molar placode and influencing cell proliferation, shape, orientation, and movement of the cells. Hence, we believe that the role of polysaccharides needs to be revisited and further analysis performed in order to understand the participation of carbohydrates in tooth development and regeneration. The role of HA in the control of molar size is very interesting and provides a tool by which tooth size could be modulated during *ex-vivo* regenerative therapies.

## Data Availability Statement

The raw data supporting the conclusions of this article will be made available by the authors, without undue reservation.

## Ethics Statement

The animal study was reviewed and approved by Comité Ético Científico para el Cuidado de Animales y Ambiente CEC-CAA, Pontificia Universidad Católica de Chile, ID number 181203001, CEBA 14024. Mouse work at King’s College London followed Home Office guidelines, with the appropriate licenses and approvals for breeding of transgenic mice in place. Culling of mice followed approved schedule one methods.

## Author Contributions

NS: performed experiments, data analysis and figures. MG-R, AW, AU, and AV: performed experiments. EC: performed experiments, discussion of data and manuscript writing. JL: performed multiphoton imaging, analysis, and discussion of data. AT and JG: did experimental design, discussion of data and manuscript writing. MG performed experiments, did experimental design, data analysis, discussion of data, manuscript writing, and figures. All authors contributed to the article and approved the submitted version.

## Conflict of Interest

The authors declare that the research was conducted in the absence of any commercial or financial relationships that could be construed as a potential conflict of interest.

## Supplementary Material

SUPPLEMENTARY FIGURE S1 HABP levels are decreased in the stellate reticulum after 4-Mu treatment. HABP levels were measured in different areas of control and 4-Mu treated placodes, showing a significant decrease of HABP detection in the stellate reticulum of 4-Mu treated germs. ^*^*p* < 0.05 (Student’s *t*-test for each tissue analyzed).

SUPPLEMENTARY FIGURE S2 Enamel knot area and M1 anterior-posterior proliferation are conserved in control and 4-Mu dental organ, but tail proliferation is increased in 4-Mu treated samples. **(A)** Enamel knot (EK)/dental organ area ratio were calculated at day 2 of culture, showing no differences between control and 4-Mu samples. **(B)** M1 was virtually divided in anterior and posterior half and pH3+ cells was quantified, no-significant differences between anterior-posterior half were found in control or 4-Mu treated germs at 3 days. **(C)** Molar tail mitotic index was calculated, showing an increase of tail proliferation in 4-Mu treated samples compared to control. Student’s *t*-test was performed. ^*^*p* < 0.05.
