# Supplementary figures and images for: Balance Between Tooth Size and Tooth Number Is Controlled by Hyaluronan
Source: Front Physiol. 2020 Aug 24;11:996. doi: 10.3389/fphys.2020.00996 (PMC7476214; doi:10.3389/fphys.2020.00996)

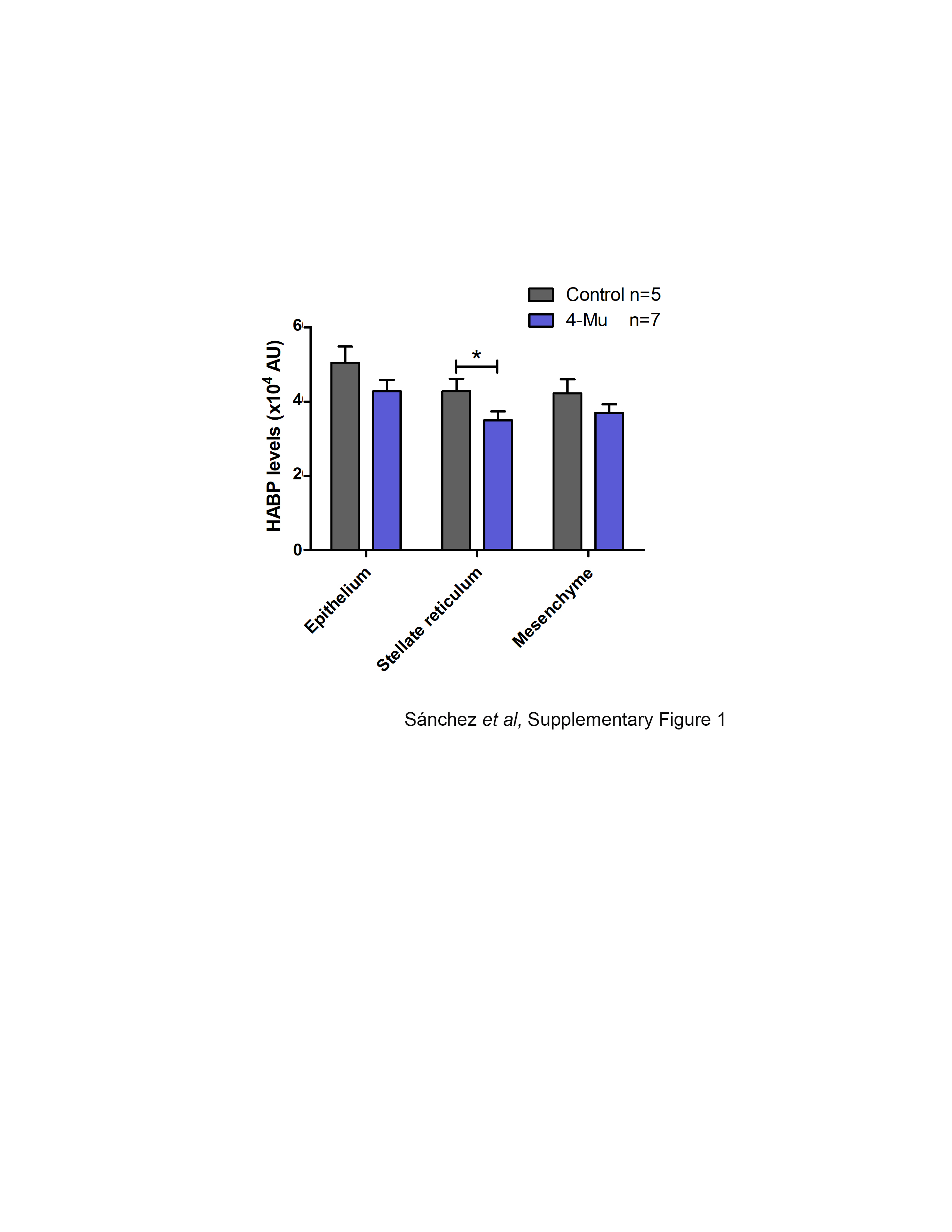

Supplement: SUPPLEMENTARY FIGURE S1 — HABP levels are decreased in the stellate reticulum after 4-Mu treatment. HABP levels were measured in different areas of control and 4-Mu treated placodes, showing a significant decrease of HABP detection in the stellate reticulum of 4-Mu treated germs. *p < 0.05 (Student’s t-test for each tissue analyzed). [file Image_1.jpeg]

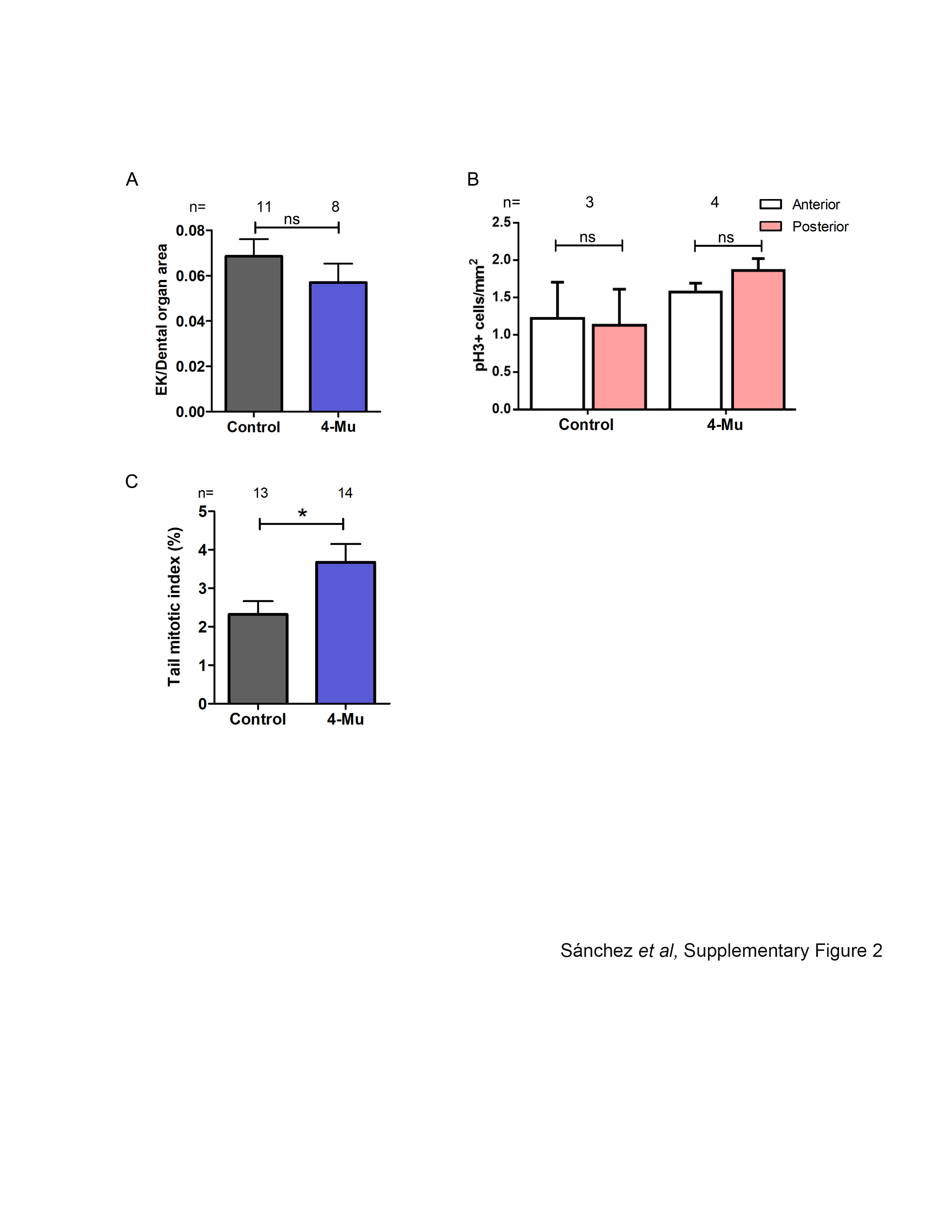

Supplement: SUPPLEMENTARY FIGURE S2 — Enamel knot area and M1 anterior-posterior proliferation are conserved in control and 4-Mu dental organ, but tail proliferation is increased in 4-Mu treated samples. (A) Enamel knot (EK)/dental organ area ratio were calculated at day 2 of culture, showing no differences between control and 4-Mu samples. (B) M1 was virtually divided in anterior and posterior half and pH3+ cells was quantified, no-significant differences between anterior-posterior half were found in control or 4-Mu treated germs at 3 days. (C) Molar tail mitotic index was calculated, showing an increase of tail proliferation in 4-Mu treated samples compared to control. Student’s t-test was performed. *p < 0.05. [file Image_2.jpeg]
